# The genomic landscape of fibrolamellar hepatocellular carcinoma: whole genome sequencing of ten patients

**DOI:** 10.18632/oncotarget.2712

**Published:** 2015-01-02

**Authors:** David G. Darcy, Rachel Chiaroni-Clarke, Jennifer M. Murphy, Joshua N. Honeyman, Umesh Bhanot, Michael P. LaQuaglia, Sanford M. Simon

**Affiliations:** ^1^ Laboratory of Cellular Biophysics, The Rockefeller University, New York, NY 10065, USA; ^2^ Division of Pediatric Surgery, Department of Surgery, Memorial Sloan Kettering Cancer Center, New York, NY 10065, USA; ^3^ Pathology Core, Department of Pathology, Memorial Sloan Kettering Cancer Center, New York, NY 10065, USA

**Keywords:** Fibrolamellar, hepatocellular carcinoma, cancer, genome, sequencing

## Abstract

Fibrolamellar hepatocellular carcinoma is a rare, malignant liver tumor that often arises in the otherwise normal liver of adolescents and young adults. Previous studies have focused on biomarkers and comparisons to traditional hepatocellular carcinoma, and have yielded little data on the underlying pathophysiology. We performed whole genome sequencing on paired tumor and normal samples from 10 patients to identify recurrent mutations and structural variations that could predispose to oncogenesis. There are relatively few coding, somatic mutations in this cancer, putting it on the low end of the mutational spectrum. Aside from a previously described heterozygous deletion on chromosome 19 that encodes for a functional, chimeric protein, there were no other recurrent structural variations that contribute to the tumor genotype. The lack of a second-hit mutation in the genomic landscape of fibrolamellar hepatocellular carcinoma makes the DNAJB1-PRKACA fusion protein the best target for diagnostic and therapeutic advancements. The mutations, altered pathways and structural variants that characterized fibrolamellar hepatocellular carcinoma were distinct from those in hepatocellular carcinoma, further defining it as a distinct carcinoma.

## INTRODUCTION

Fibrolamellar hepatocellular carcinoma (FL-HCC) is a rare, malignant liver tumor found most commonly in adolescents and young adults [1]. It is distinct from traditional hepatocellular carcinoma (HCC) in that it most commonly arises in younger individuals without a history of viral hepatitis or other signs of cirrhosis of the liver [2]. FL-HCC is often reported to have an increase of vitamin B12 receptor and less likely to have elevated alpha fetoprotein, a marker of HCC [3, 4]. Compared to HCC, studies have reached conflicting conclusions as to whether there is a difference in overall survival with FL-HCC after complete resection [5–7]. Because of the lack of defining symptoms or a specific diagnostic test, FL-HCC is often detected after it has metastasized, at which point the disease is frequently progressive and fatal. Currently there are no effective treatments for inoperable or metastatic disease.

Previous investigation into the pathogenesis of FL-HCC has mainly differentiated it from traditional HCC, with early case reports and small series defining clinical and pathologic features. Biomarkers such as elevated serum neurotensin and transcobalamin were found, but did not prove to be sensitive or specific enough for diagnosis [4, 8–10]. Further investigation into DNA ploidy and individual genes known to be mutated in traditional HCC were not found in FL-HCC, defining it as a distinct biologic entity, but one without a known pathogenesis [11]. The FL-HCC genome has been found to be relatively stable, although recurrences and metastases can exhibit more mutations [12–14]. Enlarged mitochondria in tumor cells and surrounding liver parenchyma prompted investigation into copy number aberrations and mutations of the mitochondrial genome, however, significant correlations were not found [15, 16].

Recently, next generation sequencing revealed a heterozygous deletion of approximately 400kb on chromosome 19 in all 15 samples that underwent transcriptome and whole genome sequencing [17]. Included in the deletion were eight genes whose transcript products were not significantly altered. The deletion endpoints fell within two different coding regions, creating an in-frame fusion protein with the promoter and first exon of DNAJB1, a heat shock protein, and the trailing nine exons of PRKACA, the catalytic subunit of protein kinase A. The chimeric protein retained full enzymatic activity. In the current study, we comprehensively explore the genomic landscape of FL-HCC to define the presence of other recurrent mutations in 10 patients from the same cohort. We used whole genome sequencing of paired normal and FL-HCC tissue to identify somatic mutations in coding DNA regions and other structural variants such as deletions, duplications, inversions, and translocations.

## RESULTS

The median patient age of the samples collected was 25 years old (range 17 – 46). Demographic and surgical staging information are summarized in Table 1. Regarding single-nucleotide variations (SNVs) and small insertion and deletions (indels), a median of 8957 somatic mutations was found per sample (range 5613 – 9626). This denotes a median rate of change of 2.89 mutations per megabase (range 1.81 – 3.11). The distributions of somatic changes by genomic site are found in Table 2. There was a median of 11 somatic, non-synonymous mutations per patient (range 2 – 30). Somatic base substitutions in FL-HCC were predominantly T > C or A > G transitions, followed by G > A or C > T, and the distribution of nucleotide changes are listed in Table 3. The median transition/transversion (TsTv) ratio for germline mutations was 2.06, the loss of heterozygosity was 2.15 and somatic mutations were 1.63.

**Table 1:** Patient demographics

Patient	Sex	Age at Diagnosis	AJCC Stage	Regional Lymph Nodes	Metastases	Vascular Invasion	Cirrhosis
1	M	35	4A	1	0	0	0
2	M	27	4B	1	1	1	0
3	F	31	1	0	0	0	0
4	M	46	4B	0	1	1	0
5	M	17	4A	1	0	1	0
6	F	17	1	*	0	0	*
7	F	28	4A	1	0	1	0
8	F	19	4B	*	1	1	*
9	F	23	1	0	0	0	0
10	M	22	4A	1	0	1	0

1 = Positive, 0 = Negative, * = Data absent from chart, AJCC = American Joint Committee on Cancer, 7th Edition

**Table 2:** Rate of mutations in FL-HCC per genomic site

Patient	NSC / Mb	SC / Mb	Splice / Mb	Intron / Mb	Intra / Mb
1	0.010	0.002	0.002	1.411	1.521
2	0.004	0.003	0.002	1.368	1.447
3	0.004	0.002	0.003	1.321	1.345
4	0.005	0.005	0.002	1.426	1.511
5	0.001	0.004	0.002	0.834	0.863
6	0.002	0.002	0.002	1.118	1.118
7	0.007	0.005	0.001	1.369	1.517
8	0.003	0.002	0.001	1.071	1.040
9	0.002	0.001	0.002	1.310	1.272
10	0.003	0.002	0.002	1.400	1.457

NSC = Non-synonymous coding, Mb = megabase, SC = Synonymous coding, Splice = Mutation at a transcript splice site, Intron = Mutation in intron, Intra = Mutation in intragenic region

**Table 3:** Median values for nucleotide changes

	A	C	G	T
A	*	127.5	400	109
C	138.5	*	115.5	367.5
G	373	110.5	*	157
T	125	403.5	125	*

We attempted to verify all non-synonymous, somatic mutations using Sanger sequencing (Supplemental File 1). There was a median of 6 somatic mutations verified per patient, with a range of 0–16. The distribution of verified somatic mutations, those that were unable to be sequenced due to sequence homology or repeat regions, and those calls found to be germline are listed in Table 4. Given this small cohort of 10 patients, the variant calling parameters were tuned to be more sensitive than specific and there were few somatic calls that were found to be germline upon Sanger sequencing; however, many other locations failed to amplify and sequence due to nonspecific primer binding. There were no non-synonymous, coding somatic mutations that were present in all 10 patients. There were a few mutations that were found in more than one patient, with the most frequently mutated genes being MUC4 in four patients, and GOLGA6L2, DSPP, FAM186A, HLA-DRB1, and NEFH in three patients (Table 5).

**Table 4:** Sanger sequencing verification of VarScan2-called somatic variants

Patient	Confirmed Somatic by SS	Unable to sequence	Germline by SS	% Confirmed as Somatic	% Rejected as Germline
1	16	14	1	51.61	3.23
2	8	5	1	57.14	7.14
3	5	7	1	38.46	7.69
4	12	5	3	60.00	15.00
5	0	2	4	0.00	66.67
6	1	5	2	12.50	25.00
7	15	7	1	65.22	4.35
8	3	6	0	33.33	0.00
9	4	2	6	33.33	50.00
10	7	3	3	53.85	23.08
Median	6	5	1.5	45.06	3.23

SS = Sanger sequencing

**Table 5:** Recurrent somatic, non-synonymous SNVs and indels by HUGO symbol

Gene						Patient					
	1	2	3	4	5	6	7	8	9	10	Total
MUC4	1					1	1	1			4
GOLGA6L2			1		1				1		3
DSPP	1					1	1				3
FOXO6					1	1	1				3
HLA-DRB1				1		1				1	3
PCSK5				1*			1*			1*	3
FER1L6			1*	1							2
CPS1	1*	1*									2
FAM186A	1							1			2
NEFH	1							1			2
TENM4		1*						1*			2

SNVs = Single nucleotide variation, indel = small insertion or deletion, HUGO = The Human Genome Organization, * = Verified by Sanger sequencing

To examine the cellular context of these somatic mutations, we used the Genome MuSiC suite [18]. Pathway analysis was performed using the Kyoto Encyclopedia of Genes and Genomes (KEGG) database, and filtered based on values of *p* ≤ 0.05 and FDR < 0.2 [19]. Seven pathways were affected; however, a mutation in HLA-DRB1 was common to all pathways. No other mutated genes were evident in more than one pathway, in more than one patient. There were no pathways that contained mutated genes in all 10 patients. The Significantly Mutated Genes function of MuSiC, which uses a calculated background mutation rate to report mutations that appear above baseline for each mutation category, did not return any significant results. Processing with MutSigCV, another suite designed to discover those mutations that appear in a cohort above the background mutation rate, returned no significant results [20].

**Figure 1:**
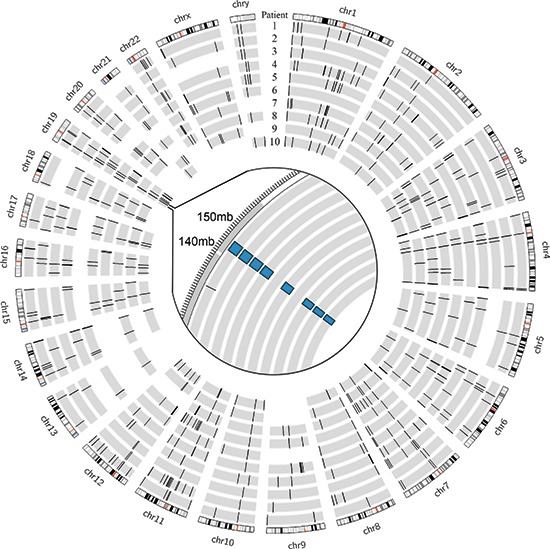
Genomic deletions in FL-HCC across 10 patients Inset with focus on chromosome 19 showing ~400kb deletion in 8/10 patients identified by DELLY.

**Figure 2:**
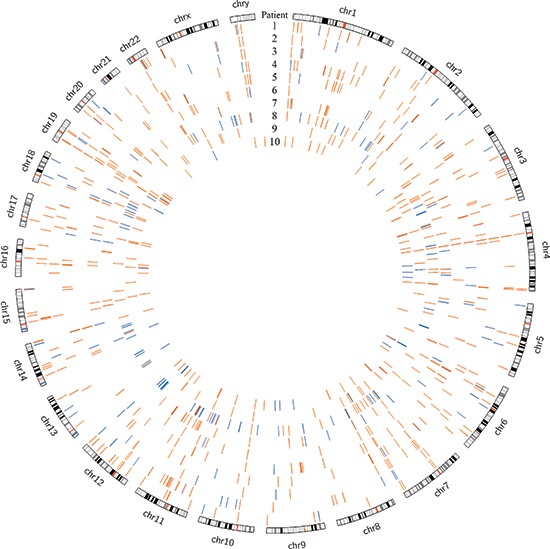
Genomic deletions in HCC (blue) and FL-HCC (orange) across ten patients each, demonstrating distinct mutational spectra

Using DELLY, we identified 3733 structural variants across all patients, with a median of 393 per patient (range 250 – 467) [21]. These were somatic mutations that were present in the tumor tissue but absent in the paired normal sample. Deletions larger than 1 kilobase, duplications, and inversions accounted for 769, 501, and 2463 events, respectively. There were no translocations identified. There were several overlapping structural variants found in multiple patients (four or more), most of which involved non-coding regions; however, only the previously described, heterozygous −400kb deletion in chromosome 19 involved coding regions and was expected to cause a functional change (Figure 1). The duplication events that encompassed coding genes were not found to be differentially expressed in the tumor compared to normal upon transcriptome analysis (data unpublished). The remaining variants fell in non-coding and repeat regions and were not expected to alter transcription or protein products. A collection of 20 hepatocellular carcinoma samples (10 primary tumor samples with paired normal liver tissue from The Cancer Genome Atlas project [TCGA]) were analyzed using the same criteria. The FL-HCC samples had a distinct mutational pattern compared to traditional HCC, most notably the recurrent heterozygous deletion in chromosome 19 (Figure 2).

## DISCUSSION

Fibrolamellar hepatocellular carcinoma was originally described in 1956 by Hugh Edmondson, a pathologist, as a previously unreported variant of traditional hepatocellular carcinoma [22]. Its clinical phenotype, typically a pediatric or adolescent patient without cirrhotic liver disease, was first described in 1980 [1]. It often presents as advanced disease, which may include regional lymphadenopathy, local invasion, and/or distant metastasis. Locally invasive or disseminated disease is relatively chemoresistant, and unless the tumor can be resected with clear margins, recurrence is common and outcomes are poor [23–25]. Thus, defining the underlying biology of this disease is essential to the development of adjuvant therapy targeting specific, causative mutations. Partially due to its low incidence, it has been difficult to collect samples for cohorts that are large enough to permit biochemical characterization. Previous studies have relied on low-resolution assays such as comparative genomic hybridization (CGH) and microarray analysis [12, 26–28].

Using fresh frozen tissue to perform whole genome analysis of SNVs, indels, and structural variants, as well as an analysis of RNA-seq, revealed the presence of a single, recurrent heterozygous deletion of chromosome 19 [17]. This genomic deletion leads to the fusion of the regulatory region and the first exon of DNAJB1 with all but the first exon of PRKACA. This functional, chimeric protein was detected in all samples and was absent from paired normal tissue. Given that this active chimeric protein was present in all tumor samples, there are two important factors to consider while analyzing the rest of the DNA. First, the significance of this chimera would be minimized if it occurred in a very high background of mutations throughout the genome. Second, there remains the possibility of other mutations or a “second hit” that may be required for tumorigenesis.

**Figure 3:**
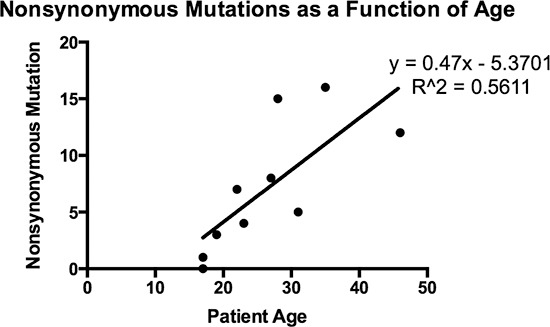
Number of somatic, non-synonymous mutations as a function of age

Analysis of the whole genome revealed that there were few other recurrent mutations, those being found in more than one patient, among the 20 samples. Along the spectrum of adult and pediatric cancers there are those with relative genomic stability, and those which have undergone chromothripsis or massive rearrangement [29]. FL-HCC has a relatively stable genome, with a single recurrent deletion found in all patients studied, and few additional mutations. These additional mutations may simply accumulate with age, although the correlation is not strictly linear, with a coefficient of determination of 0.5611 (Figure 3). This increases the significance of the one recurrent deletion found in all 10 patients, the formation of the DNAJB1-PRKACA chimera.

The most commonly mutated gene, altered in four patients, was MUC4, a transmembrane protein with a large extracellular domain that undergoes extensive glycosylation and post-translational modifications [30]. This gene has been implicated in several other epithelial cancers of the gastrointestinal system, and remains an important possible target for further investigation [30–32]. There were mutations detected by VarScan2 that we were unable to verify by Sanger sequencing, due to tandem repeats or sequence homology, a difficulty encountered when sequencing several of the MUC genes [33]. Activation of MUC4 by its external EGF domain can drive ERBB2 signaling, a transcript that was amplified in RNA-seq data from the same cohort of patients (data unpublished) [31].

The second most commonly mutated gene was GOLGA6L2, which had non-synonymous mutations in three patients. Mutations in this gene have been reported in a breast cancer sample from The Cancer Genome Atlas project; however, it has not been identified as a putative driver. The third most commonly mutated gene was DSPP, the dentin sialophosphoprotein gene. This large gene, transcribed late in the cell's replication cycle, has also been reported in 21 published studies within the Catalogue of Somatic Mutations in Cancer database [34, 35]. It is possible that these mutations arose from late replication with a depleted nucleotide pool or were sequestered from DNA repair machinery due to low transcriptional activity, as DSPP is not highly expressed in the liver [35, 36]. The remaining genes mutated in multiple samples were found in only two or three patients and consisted of the lincRNA FAM186A, as well as HLA-DRB1, FOXO6, PCSK5, FER1L6, NEFH, and TENM4. Mutations in transcription factors such as FOXO6 are intriguing, however, they appear in a minority of patients and were unable to be verified by Sanger sequencing. Discovery of additional somatic mutations and indels may require whole exome sequencing at greater depth in a larger cohort.

Carbamoyl-phosphate synthase (CPS1), confirmed to be mutated in two patients, is a mitochondrial protein involved in the urea cycle. Several case studies have shown an apparent correlation between hyperammonemia and FL-HCC [37–39]. In traditional HCC, CPS1 has been found to be suppressed by DNA methylation [40]. Whether CPS1 mutations and hyperammonemia are a correlative, causative, or contributing phenomenon has not been elucidated. Serum ammonia levels were not available in the medical records of the patients in this cohort.

Given the verified existence and functional implications of the known chromosome 19 deletion, structural variants were thoroughly investigated for additional somatic changes. Investigations of structural variants in other cancer projects have used three or more paired-end reads or mapping qualities (MAPQ) greater than 35 [41, 42]. We expanded the sensitivity of our filtering to identify structural variants with two paired-end reads, with a MAPQ greater than 20. Variants identified in the paired normal tissue by DELLY were excluded, and manual curation was used to identify additional variants in the tumor marked germline. The results were screened for germline variants by manual reference against the normal and the Database of Genomic Variants (DGV) [43]. The same filtering was applied to structural variant deletions of 20 paired HCC samples from the TCGA. Studies using immunohistochemistry and various other assays are compared to the results of VarScan2 and structural variant analysis by DELLY in Table 6.

**Table 6:** Various assays on FL-HCC compared to whole genome sequencing results

Location	Type of Change	Genes Affected	Study	FL-HCC Cohort
1q	Amplification	Numerous	Wilkens 2000 [12]	No change
7p (55, 086, 714–55, 324, 313)	Mutation Amplification	EGFR	No change Patonai 2012 [27] Polysomy without gene CNV Buckley 2005 [28]	No change No duplication events
12p (25, 357, 723–25, 403, 870)	Mutation	KRAS	No change Patonai 2012 [27] No change Muramori 2011 [58]	No change No change
17p (7, 565, 097–7, 590, 863)	Mutation	TP53	No change Honda 1998 [59]	No change
Mitochondrial DNA	Amplification Mutation	Mitochondrial genome	Decreased DNA Vivekanandan 2010 [16] No consistent mutation Vivekanandan 2010 [16]	No deletion events CPS1 mutated in 2 of 10

CNV = Copy number variation, FL-HCC = Fibrolamellar hepatocellular carcinoma

The largest contributor to structural variants was reported inversions, with 2463 among all 10 patients. This list was heavily curated to select for events found only in the tumor samples and absent from the paired normal; however, many variants had been previously reported and cataloged in the DGV. More importantly, none of the boundaries of the inversions included coding regions, greatly reducing the possibility of their contribution to a high impact mutation. All inversions remaining after filtering were heterozygous. An annotated list of the regions implicated by DELLY are found in Supplemental File 2.

Deletion events larger than 1 kilobase accounted for 769 structural variants among all patients. The known chromosome 19 deletion involving DNAJB1 and PRKACA was identified by DELLY in eight of 10 patients, and the remaining two patients did not have high-quality split reads that encompassed the breakpoints (Figure 1). The deletion was confirmed by PCR and Sanger Sequencing in all ten patients. Past studies of deletion and duplication events have shown focal or entire-chromosome arm amplifications, based on assays such as comparative genomic hybridization (CGH). We have included a summary table by Ward et al. amended with our results discovered by split read and paired-end read analysis (Table 7) [44]. While CGH techniques were able to describe large-scale changes, the split and paired-end read analysis allows for precise localization of deletion, duplication, and inversion events. No deletions were found that were precisely the same in multiple patients. No genes contained within deleted regions were found to be differentially expressed based on RNA-seq data. No identified deletions were homozygous.

**Table 7:** Summary of chromosomal losses in hepatocellular carcinoma, 5 studies of fibrolamellar hepatocellular carcinoma identified by comparative genomic hybridization studies, and results of whole genome sequencing

	HCC	FL-HCC Comparative Genomic Hybridization							FL-HCC Whole Genome Sequencing			
		Total	Total %	Individual Studies					Events	Average	Range	
# of cases	785	30		11	10	5	3	1	10			Involved Genes
1p	15%	3	10%	1	0	1	0	1	10	4.50	3–9	HNRNPCL1 (heterogeneous nuclear ribonucleoprotein C-like 1) in 3/10 HSPA7 (heat shock 70kDa protein 7) in 3/10 PPIAL4B (peptidylprolyl isomerase A (cyclophilin A)-like 4B) in 3/10 PPIAL4C (peptidylprolyl isomerase A (cyclophilin A)-like 4C) in 3/10 PRAMEF2 (PRAME family member 2) in 3/10 PRAMEF4 (PRAME family member 4) in 3/10 RNVU1-19 TTC34 (tetratricopeptide repeat domain 34) in 6/10
1q	1%	0	0%	0	0	0	0	0	8	4.13	0–8	ANKRD20A12P (ankyrin repeat domain 20 family, member A12, pseudogene) in 3/10 FAM231D (family with sequence similarity 231, member D) in 3/10 FCGR1C (Fc fragment of IgG, high affinity Ic, receptor (CD64), pseudogene) in 3/10 FCGR2C (Fc fragment of IgG, low affinity IIc, receptor for (CD32) (gene/pseudogene)) in 3/10 HNRNPCL1 (heterogeneous nuclear ribonucleoprotein C-like 1) in 3/10 HSPA7 (heat shock 70kDa protein 7) in 3/10 PPIAL4B (peptidylprolyl isomerase A (cyclophilin A)-like 4B) in 3/10 PPIAL4C (peptidylprolyl isomerase A (cyclophilin A)-like 4C) in 3/10
2p	1%	0	0%	0	0	0	0	0	8	2.38	0–4	
2q	3%	0		0	0	0	0	0	6	2.83	0–6	
3p	4%	2	0%	1	1	0	0	0	10	2.00	1–5	
3q	2%	2		0	2	0	0	0	9	3.11	0–6	MUC4 (mucin 4) in 4/10
4p	11%	2	7%	1	0	0	0	1	10	7.80	12-Mar	
4q	34%	2		2	0	0	0	0	9	2.56	0–7	
5p	2%	0	7%	0	0	0	0	0	5	1.40	0–2	
5q	8%	1	7%	1	0	0	0	0	9	1.44	0–3	
6p	1%	1	7%	0	0	1	0	0	9	2.22	0–6	
6q	15%	1	0%	1	0	0	0	0	9	2.33	0–3	ADGB (androglobin) in 3/10 BCLAF1 (BCL2-associated transcription factor 1) in 4/10
7p	1%	0	3%	0	0	0	0	0	8	1.25	0–2	
7q	3%	0	3%	0	0	0	0	0	10	4.60	2–11	DPP6 (dipeptidyl-peptidase 6) in 4/10 MUC12 (mucin 12) in 4/10 PTPRN2 (protein tyrosine phosphatase, receptor type, N polypeptide 2) in 6/10
8p	38%	5	3%	2	3	0	0	0	8	1.50	0–3	
8q	2%	0	0%	0	0	0	0	0	7	1.43	0–4	
9p	14%	2	0%	0	0	2	0	0	3	1.67	0–3	
9q	11%	1	17%	0	0	1	0	0	7	1.29	0–2	
10p	3%	0	0%	0	0	0	0	0	3	1.00	0–1	
10q	11%	0	7%	0	0	0	0	0	9	2.33	0–5	
11p	5%	2	3%	2	0	0	0	0	9	2.22	0–4	
11q	10%	1	0%	0	0	1	0	0	9	3.56	0–9	GLB1L2 (galactosidase, beta 1-like 2) in 3/10 GLB1L3 (galactosidase, beta 1-like 3) in 3/10
12p	7%	0	0%	0	0	0	0	0	6	1.83	0–5	
12q	3%	0	7%	0	0	0	0	0	8	2.88	0–7	DPY19L2 (dpy-19-like 2 (C. elegans)) in 3/10 TDG (thymine-DNA glycosylase) in 3/10
13p	0%	2	3%	2	0	0	0	0	0			
13q	26%	2	0%	0	2	0	0	0	5	1.20	0–2	
14p	0%	0	0%	0	0	0	0	0	0			
14q	11%	4	7%	2	2	0	0	0	7	4.00	0–7	ELK2AP (ELK2A, member of ETS oncogene family, pseudogene) in 3/10
15p	0%	0	7%	0	0	0	0	0	0			
15q	5%	1	0%	0	0	1	0	0	9	2.44	0–4	TYRO3 protein tyrosine kinase) in 4/10
16p	17%	3	13%	0	0	3	0	0	6	2.17	0–3	
16q	36%	1	0%	0	0	1	0	0	8	1.88	0–4	
17p	32%	1	3%	0	0	1	0	0	8	1.38	0–3	MAP2K3 (mitogen-activated protein kinase kinase 3) in 3/10 NCOR1 (nuclear receptor corepressor 1) in 3/10
17q	4%	1	10%	0	0	1	0	0	6	1.50	0–2	
18p	4%	3	3%	0	3	0	0	0	5	1.60	0–3	
18q	11%	6	3%	3	3	0	0	0	5	1.60	0–2	
19p	7%	0	3%	0	0	0	0	0	10	1.50	1–4	PRKACA, GIPC1, CD97, DDX39A, LPHN1, PKN1, ASF1B, LOC100507373, PTGER1, DNAJB1 (known genomic deletion) in 8/10
19q	4%	0	10%	0	0	0	0	0	8	2.75	0–7	
20p	2%	1	20%	0	1	0	0	0	6	1.50	0–2	
20q	1%	0	0%	0	0	0	0	0	4	1.00	0–1	
21p	0%	1	0%	0	1	0	0	0	2	1.00	0–1	
21q	9%	3	3%	2	1	0	0	0	6	1.17	0–2	
22p	0%	0	0%	0	0	0	0	0	0			
22q	6%	1	3%	1	0	0	0	0	8	2.38	0–5	ADRBK2 (adrenergic, beta, receptor kinase 2) in 3/10 CRYBB2P1 (crystallin, beta B2 pseudogene 1) in 4/10 IGLL3P (immunoglobulin lambda-like polypeptide 3, pseudogene) in 4/10 LRP5L (low density lipoprotein receptor-related protein 5-like) in 4/10
Xp	5%	1	10%	0	0	1	0	0	6	1.83	0–3	
Xq	5%	2	0%	0	0	2	0	0	6	1.50	0–3	RBMX (RNA binding motif protein, X-linked) 3/10
Yp	5%	1	3%	1	0	0	0	0	0			
Yq	6%	1	3%	1	0	0	0	0	8	3.13	0–6	
Reference	[60]			[61]	[62]	[63]	[12]	[63]				

HCC = Hepatocellular carcinoma, FL-HCC = Fibrolamellar hepatocellular carcinoma

Adapted from Ward and Waxman [44]

There were 501 duplication events remaining after structural variant filtering, results shown with previous data by Ward et al in Table 8. False positives were manually removed, such as duplications reported on chromosome 17 near the repeating KRTAP genes. The only other duplication event that involved coding regions was found in seven patients, on chromosome 22. This duplication event encompassed the genes USP41 and FAM230A, but these transcripts were not differentially expressed based on RNA-seq data (data unpublished).

**Table 8:** Summary of chromosomal gains in hepatocellular carcinoma, 5 studies of fibrolamellar hepatocellular carcinoma identified by comparative genomic hybridization studies, and results of whole genome sequencing

	HCC	FL-HCC Comparative Genomic Hybridization							FL-HCC Whole Genome Sequencing			
		Total	Total %	Individual Studies					Events	Average	Range	
# of cases	785	30		11	10	5	3	1				Involved Genes
1p	5%	0	0%	0	0	0	0	0	9	2.89	0–4	TTC34 (repeat containing protein) in 6/10
1q	57%	9	30%	1	6	0	2	0	7	1.29	0–2	C1orf186 (chromosome 1 open reading frame 186) in 4/10
2p	7%	1	3%	0	0	0	1	0	9	2.00	0–3	TPO (thyroid peroxidase) in 4/10
2q	8%	0	0%	0	0	0	0	0	6	1.17	0–2	
3p	5%	2	7%	0	1	0	1	0	1	2.00	0–1	
3q	9%	1	3%	0	0	0	1	0	8	2.63	0–4	MUC4 in 4/10
4p	6%	1	3%	0	0	0	1	0	10	7.80	0–19	
4q	2%	3	10%	0	0	2	1	0	9	2.00	0–4	
5p	14%	2	7%	1	0	0	1	0	5	3.20	0–7	
5q	11%	2	7%	0	0	1	1	0	6	2.83	0–5	CDE4D (phosphodiesterase 4D, cAMP-specific) in 3/10, PCDHB10, PCDHB16, PCDHB9 (procadherin beta) in 3/10, RAB3C, RAB3C (member RAS oncogene family) in 3/10
6p	22%	2	7%	0	2	0	0	0	7	1.57	0–2	
6q	8%	2	7%	0	0	0	1	1	5	1.40	0–2	
7p	15%	5	17%	4	0	1	0	0	3	2.33	0–4	
7q	17%	4	13%	3	0	1	0	0	9	3.22	0–8	PTPRN2 (protein tyrosine phosphatase Receptor type N2) in 5/10
8p	5%	1	3%	0	0	0	1	0	4	1.25	0–2	
8q	47%	7	23%	1	4	1	1	0	6	1.83	0–5	
9p	3%	0	0%	0	0	0	0	0	0	0.00		
9q	3%	1	3%	0	0	0	1	0	7	1.43	0–3	
10p	8%	0	0%	0	0	0	0	0	2	1.50	0–2	
10q	4%	0	0%	0	0	0	0	0	9	4.11	0–9	
11p	4%	0	0%	0	0	0	0	0	4	1.50	0–3	
11q	9%	1	3%	0	0	1	0	0	4	1.25	0–2	
12p	2%	1	3%	0	0	0	1	0	3	1.00	0–1	
12q	7%	1	3%	0	0	0	1	0	4	1.00	0–1	
13p	0%	0	0%	0	0	0	0	0	0	0.00		
13q	7%	1	3%	0	0	0	0	1	6	1.00	1.00	
14p	0%	0	0%	0	0	0	0	0	0	0.00		
14q	4%	0	0%	0	0	0	0	0	4	1.50	0–2	
15p	0%	1	3%	0	1	0	0	0	0			
15q	5%	1	3%	0	1	0	0	0	8	1.88	0–3	
16p	3%	3	10%	1	2	0	0	0	8	1.75	0–4	
16q	2%	2	7%	1	1	0	0	0	7	1.86	0–3	
17p	3%	1	3%	1	0	0	0	0	5	1.00	0–1	
17q	22%	0	0%	0	0	0	0	0	7	1.86	0–2	KTRAP2-1, KTRAP2-2 (keratin associated protein) in 3/10
18p	6%	1	3%	0	0	0	1	0	1	1.00	0–1	
18q	5%	1	3%	0	0	0	1	0	3	1.00	0–1	
19p	5%	3	10%	3	0	0	0	0	3	1.67	0–2	
19q	10%	2	7%	1	1	0	0	0	5	1.00	0–1	
20p	15%	0	0%	0	0	0	0	0	2	1.50	0–2	
20q	19%	2	7%	1	1	0	0	0	5	1.00	0–1	CDH4 (cadherin 4, type 1, R-cadherin) in 3/10
21p	0%	0	0%	0	0	0	0	0	1	1.00	0–1	
21q	2%	0	0%	0	0	0	0	0	2	1.50	0–2	
22p	0%	0	0%	0	0	0	0	0	0			
22q	3%	0	0%	0	0	0	0	0	7	1.71	0–2	USP41 (predicted), FAM230A
Xp	11%	3	10%	1	1	0	1	0	6	1.33	0–2	
Xq	15%	3	10%	1	0	0	1	1	5	2.00	0–4	
Yp	2%	0	0%	0	0	0	0	0	1	1.00	0–1	
Yq	2%	0	0%	0	0	0	0	0	7	2.86	0–6	
Reference	[60]			[61]	[62]	[63]	[12]	[63]				

HCC = Hepatocellular carcinoma, FL-HCC = Fibrolamellar hepatocellular carcinoma

Adapted from Ward and Waxman [44]

There is a distinctly different pattern of mutation and structural variations seen when comparing our FL-HCC data to studies of traditional HCC. Whole genome sequencing from twenty samples of HCC and paired normal tissue, obtained from TCGA, were processed for structural variant deletion events in the same manner as the FL-HCC data (sample IDs listed in Supplemental File 3). The recurrent chromosome 19 deletion was absent from the HCC data, and no other deletion events were recurrent in both FL-HCC and HCC (Supplemental File 4). Many well-powered studies have defined the genetic and molecular characteristics of HCC; the most common mutations affect the WNT/beta-catenin, p53, and PI3k/Ras pathways [45, 46]. Individual genes found to be mutated in several studies include TP53, ERRFI1, CTNNB1, AXINI, CDKN2A, and more recent studies have highlighted miRNA and lncRNA as potential oncogenic factors [47, 48]. None of these mutations were consistently found in our FL-HCC cohort. Additionally, studies regarding intratumoral heterogeneity have found a great deal of clonal variation and a variety of mutational patterns in HCC [49]. Unfortunately, for development of HCC treatments, most of these mutations are loss of function, and are not easily targetable for therapeutic intervention. In stark contrast, FL-HCC seems to have a relatively consistent mutational spectrum, with low tumor heterogeneity, a paucity of somatic mutations overall, and an active chimeric protein in all samples studied thus far [50].

In summary, we explored the genomic landscape of FL-HCC in a cohort of 10 patients using primary liver tumors and matched normal tissue. The demographic information for these 10 patients was typical for patients presenting with FL-HCC, and all samples were verified and extracted with the goal of highest quality sequencing output. We searched broadly for a mutation or structural change that could possibly contribute to the pathogenesis of FL-HCC, in addition to the active chimeric protein kinase DNAJB1-PRKACA. Analysis of the single nucleotide variants and small insertions and deletions did not reveal a consistent, deleterious mutation in all 10 patients. Several proteins involved in cancer pathways had mutations in more than one patient, the most common being MUC4, GOLGA6L2, DSPP, FOXO6 and HLA-DRB1. Analysis of the structural variants found a variety of deletions, duplications, and inversion events, however only one mutation involved breakpoints within coding regions, the previously discovered chromosome 19 mutation. Other variants will require further investigation to elucidate which may contribute to the FL-HCC phenotype via dysregulation of transcription factors or promoter regions. Furthermore, analysis of more samples will be required to determine if any of these alterations are correlated with patient outcomes. Although there were other somatic mutations and structural variations, no other changes were found in all patients, offering further support for the chimeric DNAJB1-PRKACA kinase as necessary and sufficient to the oncogenesis of FL-HCC. This genomic data will provide a framework for future studies, and has clearly defined fibrolamellar hepatocellular carcinoma as a unique pathologic entity, distinct from traditional hepatocellular carcinoma.

## METHODS

With IRB approval (Rockefeller IRB SSI-0797 and Memorial Sloan Kettering IRB Protocol # 13-010), 20 snap-frozen tissue samples were collected from 10 patients, consisting of primary liver tumor and adjacent normal parenchyma. A pathologist specializing in liver tumors used H&E microscopy to verify that all tumor samples were FL-HCC and that normal samples were free of cirrhosis. Macrodissection was performed to exclude inflammatory cells in tumor and normal tissue, as well as to remove the stromal bands in the tumor tissue where possible. All tumor samples contained minimal necrosis and > 80% tumor cells. There were no samples that contained mixed HCC and FL-HCC, and all normal samples were free from tumor infiltration. Whole genomic DNA was extracted from the macro-dissected tissue using DNeasy Blood and Tissue Kit (Qiagen, Hilden, Germany). Manufacturer's protocols were used to prepare the libraries using the Illumina TruSeq PCR free kit (Illumina, San Diego, US). Samples were sequenced on the Illumina HiSeq 2500, with five pairs of tumor and normal samples in rapid mode at 2 × 150 bp paired-end, and five pairs in high output mode at 2 × 100bp paired-end. The average base coverage for tumor and normal samples was of 60-fold and 30-fold, respectively. The GRCh37 reference genome was using for all subsequent steps. Alignment, recalibration, and deduplication were carried out using BWA [51], Picard Tools [52] and The Genome Analysis Toolkit [53] using standard best-practice guidelines. SNVs and indels were detected using VarScan2 [54], and structural variants were identified using DELLY 0.5.3 [21] using paired-end and split-read mapping. SNV and indel gene annotation was performed with Oncotator [55]. Structural variant annotation was performed using BedTools and UCSC Genome Browser data sets [56, 57]. The perl and python scripts included with VarScan2 and DELLY were used along with manual filtering to filter false positive and germline events. The Database of Genomic Variants database used was updated in July of 2013 [43]. The whole genome sequencing data for this project are available at the Database of Genotypes and Phenotypes (http://www.ncbi.nlm.nih.gov/gap/?term=phs000709).
